# Changes in Redox Signaling in the Skeletal Muscle with Aging

**DOI:** 10.1155/2019/4617801

**Published:** 2019-01-17

**Authors:** Péter Szentesi, László Csernoch, László Dux, Anikó Keller-Pintér

**Affiliations:** ^1^Department of Physiology, Medical Faculty, University of Debrecen, Debrecen H-4002, Hungary; ^2^Department of Biochemistry, Faculty of Medicine, University of Szeged, Szeged H-6720, Hungary

## Abstract

Reduction in muscle strength with aging is due to both loss of muscle mass (quantity) and intrinsic force production (quality). Along with decreased functional capacity of the muscle, age-related muscle loss is associated with corresponding comorbidities and healthcare costs. Mitochondrial dysfunction and increased oxidative stress are the central driving forces for age-related skeletal muscle abnormalities. The increased oxidative stress in the aged muscle can lead to altered excitation-contraction coupling and calcium homeostasis. Furthermore, apoptosis-mediated fiber loss, atrophy of the remaining fibers, dysfunction of the satellite cells (muscle stem cells), and concomitant impaired muscle regeneration are also the consequences of increased oxidative stress, leading to a decrease in muscle mass, strength, and function of the aged muscle. Here we summarize the possible effects of oxidative stress in the aged muscle and the benefits of physical activity and antioxidant therapy.

## 1. Introduction

With improved life quality conditions and the availability of treatments, life expectancy and consequently the number of elderly in the population have increased [1]. This change in population composition places increasing emphasis on the treatment of chronic, noncommunicable diseases as they have become major causes of death and disability worldwide, thus driving the need to understand the mechanism of aging and find treatments for age-related diseases [2].

**Figure 1:**
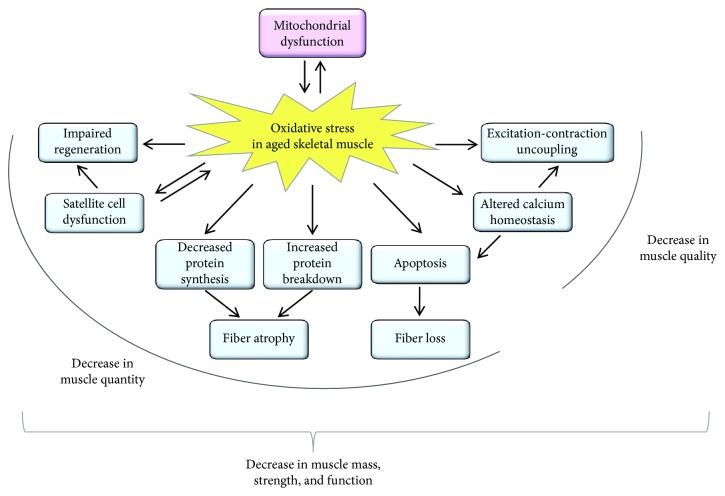
Schematic summary of the effects of oxidative stress in the aged skeletal muscle. The age-related increase in oxidative stress can result in mitochondrial dysfunction, and the dysfunctional mitochondria can further generate reactive oxygen species. The increased oxidative stress can lead to a decrease both in muscle quality and in muscle quantity. As a consequence of the increased oxidative stress, excitation-contraction uncoupling, altered calcium homeostasis, apoptosis-mediated fiber loss, atrophy of the remaining fibers, dysfunction of the satellite cells (muscle stem cells), and impaired muscle regeneration can be observed in the aged muscle leading to a decrease in muscle mass, strength, and function.

The skeletal muscle is the largest organ in the body comprising ~40% of its mass. It plays fundamental roles in movement, posture, and energy metabolism. The loss of skeletal muscle mass and function with age can have a major impact on quality of life and results in increased dependence and frailty. Age-related decline of skeletal muscle function (sarcopenia) results in strength loss [3]. This loss stems from two major sources, reductions in muscle mass (i.e., quantity) and decrease in its intrinsic capacity for producing force (i.e., quality). Both can be the consequence of several factors (Figure 1), including oxidative stress that is the result of the accumulation of reactive oxygen and nitrogen species (ROS/RNS). The free-radical theory of aging was established more than 60 years ago [4] and has become one of the most studied theories to have been proposed. It is now accepted that this theory and its various spin-offs cannot alone explain the aging process [5, 6]. Nevertheless, huge amounts of data indicate that ROS-mediated aging phenotypes and age-related disorders exist [7, 8].

During physiological homeostasis the overall oxidative balance is maintained by the production of ROS/RNS from several sources and their removal by antioxidant systems, including endogenous or exogenous antioxidant molecules. At physiological concentrations ROS/RNS play essential roles in a variety of signaling pathways. There is an optimal level of ROS/RNS to sustain both cellular homeostasis and adaptive responses, and both too low and too high levels of ROS/RNS are detrimental to cell functions [9]. The skeletal muscle consumes large quantities of oxygen and can generate great amounts of ROS and also reactive nitrogen species. Mitochondria are one of the most important sources of ROS in the skeletal muscle; furthermore, NADPH oxidase (NOX) [10], xanthine oxidase [11], and phospholipase A2 (PLA2) [12, 13] are also involved in ROS production.

The origin of the increased ROS production and oxidative damage is mitochondrial dysfunction with aging [14], caused by age-related mitochondrial DNA mutations, deletions, and damage [15], as well as the impaired ability of muscle cells to remove dysfunctional mitochondria [16]. Oxidative phosphorylation impairment can lead to decreased ATP production and further generation of ROS [4]. Interestingly, aging is associated not only with an increase in oxidative damage but also with an upregulation of antioxidant enzymes in the skeletal muscle [9]. Furthermore, the iron content of the mitochondria in the skeletal muscle increases with aging, amplifying the oxidative damage with the generation of ROS [17]. Increased ROS production, mitochondrial DNA damage, and mitochondrial dysfunction was observed in aged muscles [18–20].

The skeletal muscle is highly plastic and shows several adaptations towards mechanical and metabolic stress [21, 22]. Oxidative stressors, like ROS, have long been taken into account as harmful species with negative effects in the skeletal muscle [23]. Proteins such as biomolecules are frequently affected by oxidation; thus, elevated ROS levels can cause reversible or irreversible posttranslational modification of cysteine, selenocysteine, histidine, and methionine. Oxidative posttranslational modifications of proteins are characteristic in the aged muscle, such as carbonylation which alters protein function [24]. The oxidative capacity of muscles is strongly associated with health and overall well-being. Enhanced oxidative capacity in the skeletal muscle protects against several pathological phenomena (insulin resistance, metabolic dysregulation, muscle loss with aging, and increased energetic deficits in myopathies) [25, 26]. These protective effects are largely associated with enhanced mitochondrial function and elevated numbers of mitochondria, which can protect against cellular stress.

Given the rapidly aging population, it is essential to better understand the development, progression, prevention, and treatment of age-related muscle diseases. The aim of this review is to discuss the possible effects of age-related oxidation on the skeletal muscle and highlight the benefits of physical activity and intake of antioxidant compounds to protect from oxidative stress.

## 2. Oxidative Stress and EC-Coupling Machinery in Aging

**Figure 2:**
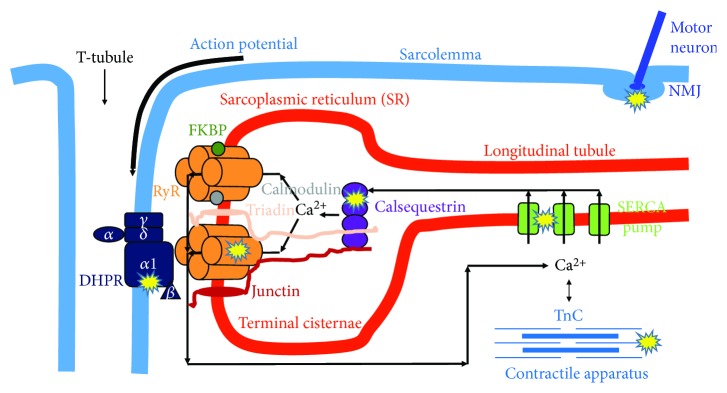
Possible actions of age-related oxidative stress reducing skeletal muscle contraction. Accumulation of reactive oxygen and nitrogen species in the aged muscle results in protein modification and/or damage that could reduce muscle quality by altering muscle fiber activation at the neuromuscular junction (NMJ), excitation-contraction (EC) coupling (DHPR, RyR, SERCA, calsequestrin), and cross-bridge cycling within the myofibrillar apparatus. DHPR: dihydropyridine receptor; FKBP: FK506 binding protein; RyR: ryanodine receptor; SERCA: sarco/endoplasmic reticulum Ca^2+^ pump; TnC: troponin-C.

Ca^2+^, as a second messenger, is necessary for muscle contraction. Ca^2+^ can originate from the extracellular space (the heart and smooth muscle) and from the intracellular store of the sarcoplasmic reticulum (SR) (in the skeletal muscle exclusively from SR). Excitation-contraction (EC) coupling, the steps from the trigger action potential to the development of force, starts with the activation of the voltage sensor dihydropyridine-sensitive, L-type Ca^2+^ channels (DHPRs). These can open the Ca^2+^ release channel ryanodine receptor (RyR) of SR [27]. The released Ca^2+^ freely diffuses into the intracellular space and, after binding with troponin-C (TnC), initiates muscle contraction. During relaxation, the Ca^2+^ is taken up by the sarco/endoplasmic reticulum calcium pump (SERCA) into the SR [28]. If any step in the EC-coupling pathway is disrupted, the voltage-induced Ca^2+^ release from SR will be deficient and less cytoplasmic Ca^2+^ will be available to bind with TnC (Figure 2).

EC coupling has a unique structure in muscle fibers, called calcium release units (CRUs). They are formed by two membrane systems: the transverse- (t-) tubule, where the trigger action potential (depolarization) from the sarcolemma goes into the fiber, and the calcium store SR terminal cisternae [29]. In a fully developed skeletal muscle fiber, a central t-tubule usually forms junctions with two SR forming a triad. The voltage sensor DHPRs localized in the t-tubule membrane [30] are in direct connection with the closely apposed calcium release channel RyRs in the SR membrane.

### 2.1. RyR

Mammalian RyR has three isoforms, which were originally identified in the skeletal muscle (RyR1), in the heart muscle (RyR2), and in the brain (RyR3). It is now known that some tissues express all three mammalian RyR isoforms [31–33]. Several cellular compounds (e.g., ATP, HCl, Ca^2+^, and Mg^2+^), specific proteins (phosphatases and kinases), and endogenous oxidative species can regulate RyR functions [34]. Abramson and Salama [35] were the first to propose a redox-dependent gating model of RyR, in which the channel pore opens after the oxidation and closes after the reduction of critical sulfhydryl moieties within the RyR complex. Gating transitions of the RyR channel are extremely fast; the open state usually lasts no longer than a few milliseconds; thus, this hypothesis has been called into question because of its slow kinetic [36]. Additionally, isolated RyR1 reconstituted in an artificial lipid bilayer functioned similarly independently of the presence of cofactors to maintain the catalytic transfer of electrons [37].

Another possibility to control RyR1 gating is the transmembrane redox potential of SR. In healthy mammalian cells, the redox potential of the cytosol is approximately -230 mV [38]. The majority of redox buffers within the cytosol of a muscle cell are based on the relative concentration of oxidized (GSSG) and reduced (GSH) glutathione or NADH and NAD^+^. In different nonmuscle cells, GSSG and GSH transporters have been found across the ER membrane [38, 39]. These transporters play an essential role in establishing and maintaining the membrane redox potential gradient. It was shown that glutathione transport across SR/ER membranes is very fast and correlates with the expression of RyR1 in terminal cisternae [40]. These findings imply the presence of one or more transmembrane redox sensors in the RyR1 channel.

To study the redox regulation of RyR1 channel activity, Feng et al. [41] used artificial lipid bilayer membranes and precisely controlled the redox state by adjusting the [GSSG]/[GSH] ratio to change redox potentials on both the lumenal and cytoplasmic sides of the reconstituted channel. Redox sensing may represent a widespread mechanism by which RyR1 channels respond to local changes in transmembrane redox potential. As mentioned above, disulfide bond formation (sulfhydryl oxidation) in RyR1 usually takes place in the oxidizing environment of the SR lumen, not in the reducing environment of the cytosol.

Pessah et al. [42] demonstrated that RyR1 channel gating was accompanied by changes in the microenvironment of hyperreactive Cys residues. It was assumed that the localized redox potential could influence the domain with the redox sensor, which might change the stability of the closed state. This means that the closed but not the open conformation of RyR1 senses redox changes. In this framework, the rapid gating transitions of RyR1 would not coincide with oxidation and reduction of disulfide bonds, and local changes in the redox environment would influence the overall operation of the channel.

With advancing age, RyR becomes increasingly oxidized and nitrosylated, which leads to leaky release channels. RyR1 from aged mice was shown to be more oxidized and cysteine-nitrosylated compared to that from young animals. Furthermore, these RyR channels lacked the stabilizing subunit FKBP12. Treating aged mice with the small molecule rycal drug S107 stabilized binding of FKBP12 to RyR1 reducing intracellular Ca^2+^ leakage, enhancing Ca^2+^ release from SR, decreasing ROS, and improving muscle exercise [43]. Similarly, increased Ca^2+^ leakage from the SR, primarily through the RyRs, was found in type I fibers of aged humans, and a reducing treatment with dithiothreitol inhibited RyR Ca^2+^ leakage, thus increasing net SR Ca^2+^ accumulation [44]. Other evidence of partially defective SR in the aged muscle is the decreased frequency of spontaneous Ca^2+^ release (spark) through RyR, observed by Park et al. [45] and the authors of this review (unpublished data).

The expression of RyR also changes with age. Unpublished data of the authors of this review showed reduced RyR expression in aging mice. The whole tetramer was almost completely absent in the EDL muscle of old animals, and only a smaller amount of degraded RyR was found. Interestingly, this was not the case in mice that did voluntary exercise throughout their entire life.

### 2.2. RyR-Associated Proteins

A lot of studies have investigated the redox dependence of accessory proteins of RyR1, which contribute to the tight regulation of channel activity in the mammalian skeletal muscle. These proteins include the voltage sensor skeletal dihydropyridine receptor (L-type Ca^2+^ channel), calmodulin, triadin, junctin, FKBP12 (12 kDa FK506 binding protein), and calsequestrin in the SR lumen [34, 46].

To date there is no evidence that triadin and junctin have any role in the redox regulation of RyR1. On the other hand, reactive sulfhydryl groups within RyR1 channels have been shown to help the binding of calmodulin, and functional responses of calmodulin to RyR1 may be redox regulated [47]. It was proposed that probably more than one class of sulfhydryl residues within the RyR1 channel complex suffer chemical modification, each contributing to a specific function. This question is still open because of the structural complexity of RyR1 and its associated proteins.

Calsequestrin-1 is a high-capacity Ca^2+^ buffer, localized in the lumen of SR in close proximity to RyR1. It has been demonstrated that nNOS and NOX2 also colocalize with RyRs at the triad junctions, and the latter generate ROS, which stimulate Ca^2+^ release from the SR through RyR1 [48]. Recently, it was hypothesized that in muscle fibers lacking Calsequestrin-1, the close positioning of either nNOS or NOX2 to RyR1 and the Ca^2+^-dependent activation of nNOS could be the consequence of increased production of ROS and RNS. This could finally lead to nitrosylation and glutathionylation of specific cysteine residues causing oxidative modifications that further increase the probability of leaky RyR1 channels [49].

RyR1 has four subunits to bind the small FK506 protein (FKBP12) [50]. FKBP12 associates mainly with the skeletal muscle isoform to regulate RyR1 function. Pharmacological removal of FKBPs causes uncoupling of RyR1 ion channels from their neighbors and thus activates Ca^2+^ release from SR [51]. A recent study shows that the 1,4 benzoderivative S107 binds to multiple RyR1 sites with low affinity and stabilizes the RyR1-FKBP12 complex depending on the redox state of the calcium channel [52].

### 2.3. DHPR

DHPR is located in the t-tubules and plays a role in EC coupling as the voltage sensor triggering Ca^2+^ release from the SR after an action potential. More than 20 years ago, Delbono et al. [53] recorded a significant reduction of maximum charge movement and L-type calcium current in muscle fibers from biopsies of 65-75-year-old patients. This was accompanied with decreased Ca^2+^ release from the SR. Just a few years later it was shown that ROS may also target DHPRs, since ROS alter the dynamics of muscle K^+^ contractures [54]. A later study by the same research group using the mammalian diaphragm demonstrated an increase in tension after antioxidant application that is clearly dependent on DHPR function [55]. These results support the hypothesis that the DHPR redox state and RyR function are modulated in an interactive manner and modify contractility.

It was also shown that the expression of the *α*1 subunit of DHPR decreases with age and this is associated with the loss of skeletal muscle strength [56]. These findings were amplified by the fact that DHPR expression levels can be regulated by different mechanisms, independently from gene transcription or mRNA expression. In a very recent study, a novel finding was reported that cytoplasmic-located fast skeletal muscle troponin T3 (TnT3) regulates DHPR expression in skeletal muscle fibers and calpain-induced cleavage of TnT3 is associated with DHPR downregulation in aged mice [57]. The reduced DHPR expression with aging increases the number of uncoupled RyR1s and, thus, decreases SR Ca^2+^ release which leads to EC uncoupling and finally decreased force production.

### 2.4. SERCA

The sarco/endoplasmic reticulum Ca-ATPase (SERCA) is the calcium pump that uptakes Ca^2+^ from the cytosol to the SR during muscle relaxation. It has an important role in maintaining the resting intracellular Ca^2+^ concentration (around 100 nM). Evidence for NO inhibition of the Ca-ATPase was observed in the rabbit skeletal muscle, where sustained contractions led to significant (40–50%) inactivation of the pump [58]. One possible explanation could be the reactions with critical SH groups, since peroxynitrite treatment of Ca-ATPase from the rabbit skeletal muscle was correlated with oxidative and nitrosative modifications of cysteines at several positions, of which one was deemed responsible for enzyme inhibition [59]. Recently it was shown that SERCA1 is reversibly regulated via NO-dependent S-glutathiolation of specific cysteine residues which are embedded within the transmembrane domains of the pump. Some specific amino acid peroxides react selectively with a subset of cysteine residues of SERCA1, representing one of the targets for NO-dependent S-glutathiolation [60]. In a parallel study it was also demonstrated that antioxidant treatment affects intracellular Ca^2+^ concentration, increasing the maximum rates of ATP hydrolysis and uptake of Ca^2+^ by SERCA in the diaphragm [61].

The 53 kDa isoform of sarcalumenin, the major luminal glycoprotein associated with SERCA, was found to be downregulated in the aged human muscle [62]. Interestingly this was accompanied with the upregulation of Calsequestrin-1. In a recent study on humans, it was shown that these changes were reversed after 9 weeks of training by electrical stimulation [63] of the *vastus lateralis* muscle of sedentary senior volunteers. The decreased active SERCA, and thus insufficient SR Ca^2+^ content, can also be explained by the findings of Boncompagni et al. [64]. Their electron microscopic study proved the presence of SERCA and Calsequestrin-1-rich tubular aggregates in the aging mouse skeletal muscle. They hypothesized that polymerization of SERCA induces its inactivity and this decreases the Ca^2+^ uptake capacity of SR. Similarly, the accumulated inactive Calsequestrin-1 in tubular aggregates is missing from SR and leads to reduced Ca^2+^ storage capacity.

## 3. The Effects of Age-Dependent Structural Changes in the Skeletal Muscle

It has been suggested by Renganathan and colleagues [56] that an uncoupling between DHPR and RyR1 in the CRUs (insufficient transmission of the sarcolemmal depolarization to the calcium release channel) with aging is one of the major determinants of the progressive decline in muscle strength. This has been supported by transmission EM studies [65], which show a progressive disarrangement of triads in the aging human skeletal muscle. This results in a drastic reduction in the overall number of CRUs available for releasing Ca^2+^ to initiate the sliding of contractile filaments and generate force. Notwithstanding that the total number of CRUs is decreased, on average by more than 50% in the aging muscle, the decrease in the total amount of both DHPR and RyR1 was less, because the decrease in the total number of SR/t-tubule junctions is accompanied by an increase in the average size of RyR clusters which compensate for the loss of triads.

Besides Ca^2+^, ATP is also necessary to generate force, as well as for relaxation in the skeletal muscle. The main source of ATP is mitochondria. It was shown that mitochondria and CRUs are functionally linked to each other via ROS- and Ca^2+^-mediated cross-talk [66]. Furthermore, these two organelles are structurally connected by tethers, which promote proximity and sufficient calcium signaling [67]. In the aged muscle, not only the ultrastructure, density, and disposition of mitochondria and CRUs but also their reciprocal associations are altered. The density of CRUs and mitochondria is decreased in the aged muscle, with an increased number of damaged mitochondria and mitochondria misplaced from their normal triadic position. A significant reduction in CRU-mitochondria pair density and their tethering was also observed in aged mice. These changes were accompanied with increased oxidative stress and with decreased mitochondrial Ca^2+^ uptake and SR Ca^2+^ release [68]. These wrong direction changes in the skeletal muscle can be prevented by regular exercise. The number of mitochondria is higher in athletic than in sedentary seniors, and furthermore, the number of CRU-mitochondria pairs is three times higher in senior sportsmen than in sedentary individuals. Since the correct association between CRUs and mitochondria is necessary for efficient ATP production, this can explain the significantly superior muscle performance in lifelong exercising seniors [68]. Similar results were obtained with mice that had access to running wheels for the second part of their lives (from 1 to 2 years of age) [69]. The authors of these studies concluded in their results that the huge age-dependent decrease affecting EC-coupling apparatuses and mitochondrial functions in the skeletal muscle of humans and mice can be partly associated with inactivity in old age.

## 4. Oxidative Stress and Satellite Cell Dysfunction with Aging

**Figure 3:**
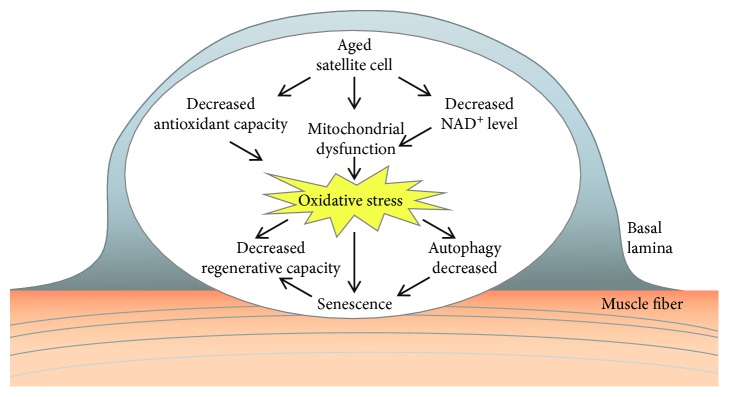
Age-related alterations in satellite cells. Mitochondrial dysfunction and decreased antioxidant capacity of aged satellite cells can lead to increased oxidative stress. The satellite cell dysfunction results in decreased regenerative capacity of the muscle. As a consequence of increased oxidative stress, a decrease in autophagy can lead to senescence.

The skeletal muscle has the remarkable ability to regenerate in response to injury. This regenerative capacity is due to the muscle stem cells (MuSCs), also called satellite cells that reside between the muscle fiber and its surrounding basal lamina [70]. The satellite cells are mitotically and physiologically in a quiescent state (a G0 reversible arrest state) in the healthy muscle and express the Pax7 transcription factor. They are stimulated upon muscle injury to enter the cell cycle and proliferate extensively and form myoblasts that will subsequently differentiate and fuse to form muscle fibers. The differentiated myocytes are capable of fusing together and, with the preexisting myofibers, restore the muscle tissue. A small subset of the expanding satellite cells does not commit to terminal differentiation but self-renews to restore the quiescent satellite cell pool for further needs [71]. The regenerative function of satellite cells declines with age [72, 73] (Figure 3). At advanced geriatric age, this decline is maximal owing to transition from a normal quiescence into an irreversible senescence (a G0 irreversible arrest).

The age-related deficits in muscle regeneration have been linked to changes in the satellite cell environment (such as inflammatory status) and/or satellite cell-intrinsic mechanisms [74]. Both the number and the functionality of satellite cells decrease with age [75–79], switching from quiescence to a senescent state [76]. The satellite cells are unequally distributed among the different fiber types and differ between muscles. In the rat extensor digitorum longus (EDL) muscle, satellite cells are observed most frequently on type IIA fibers and at approximately equal frequencies on type IIB and type I fibers [80]. Interestingly, the soleus contains a considerably higher percentage of satellite cells than the EDL [80]. In the young adults, satellite cell content did not differ between type I and type II muscle fibers [81]. Aging is associated with a switch from fast to slow fiber type [82]. The abundance of resident satellite cells declines with age in myofibers from both fast- and slow-twitch muscles in mice [83], and a decrease in FGF signaling as a possible limiting factor of satellite cell function during muscle aging has been identified [83]. In contrast, satellite cell content is reported to be specifically reduced in type II skeletal muscle fibers in the elderly, but not in type I fibers [84]. This decline in satellite cell content might be an important factor in the etiology of type II muscle fiber atrophy, which accompanies the loss of skeletal muscle with age [81, 84].

The function of satellite cells is altered by oxidative stress with aging. In the aged muscle, the satellite cells exhibit a reduced capacity to proliferate and self-renew. The decrease in the self-renewing muscle stem cell pool can lead to decreased regenerative capacity of the muscle. The quiescent satellite cells have a low metabolic rate and display only a few active mitochondria and therefore are exposed to low levels of oxidative stress [85]. Gene expression studies have also indicated differences in the transcriptional profile of aged versus young satellite cells, e.g., changes in genes associated with mitochondrial function [86]. The ROS production was higher in isolated satellite cells from the aged muscle [87]. Furthermore, a decline in antioxidant capacity in satellite cells was also observed with age, diminishing satellite cell function with increased ROS levels [88]. It was reported that the antioxidant activity of catalase and glutathione transferase is reduced in aged satellite cells [89]. Several redox-dependent signaling pathways are deregulated in aged satellite cells; decreased Notch [90], increased Wnt (wingless/integrated) [91], increased p38/MAPK (mitogen-activated protein kinase) [77], and JAK-STAT3 (Janus kinase-signal transducer of activation) [92] signaling were observed.

Mitochondrial dysfunction can result from decreased NAD^+^ (nicotinamide adenine dinucleotide) levels of the cells. Stem cells are thought to rely predominantly on glycolysis to yield energy, decreasing NAD^+^ concentration [93]. The reduction of the cellular NAD^+^ level and its effect on mitochondrial activity was shown to be a pivotal switch to modulate satellite cell senescence [78]. Treatment with the NAD^+^ precursor, nicotinamide riboside, induced the mitochondrial unfolded protein response and synthesis of prohibitin proteins, rejuvenated the muscle stem cells in aged mice, and enhanced life span [78].

The activities of the ubiquitin-proteasome system, autophagy, and chaperones appear to decline with age [94]. During myogenesis and regeneration, an increase in protein synthesis and removal of misfolded proteins can be observed [73]. Oxidative stress can influence muscle satellite cells by altering their protein homeostasis. Basal autophagy is essential to maintain the stem cell quiescent state [95]. Autophagy was shown to be essential to maintain the stemness of satellite cells by preventing the senescence caused by mitochondrial dysfunction and oxidative stress associated with aging [95].

## 5. Age-Related Muscle Loss and Oxidative Stress

Sarcopenia, the age-associated generalized and progressive reduction in muscle mass, increases the susceptibility to muscle injury, serious falls, obesity, and diabetes [96], predicting frailty, disability, poor quality of life, and mortality in the elderly [97–101]. The prevalence of low muscle mass is estimated between 8 and 40% depending on the population studied and the methods used to identify sarcopenia; it ranges from 15% at 65 years to 50% at 80 years [100, 101]. Progressive muscle loss starts at approximately the age of 40 years; it is estimated at about 8% per decade until the age of 70 years and then it increases to 15% per decade [102]. Reduction in muscle mass is usually combined with an increase in body fat mass; the accumulation of fat can be observed within the muscle fibers. The high levels of ROS in the aging muscle can induce the transition of satellite cells into an adipogenic phenotype. This muscle-to-fat transition can explain the increased intramuscular adipose tissue associated with sarcopenia [103, 104].

Age-related muscle atrophy was shown to be associated with a decrease in the total number of muscle fibers and a simultaneous decrease in the size of the individual fibers. It was reported that age-related muscle loss in rodents [105] and humans [82] can occur due to the loss of muscle fibers and a decrease in the cross-sectional area of the remaining fibers. Several factors were reported to contribute to muscle atrophy with aging. The role of reduced protein synthesis, declines in neural function, hormonal deficits, chronic low-grade inflammation, loss of mitochondrial function, nuclear apoptosis, reduced function of satellite cells, and oxidative stress was reported [96, 106].

A relationship was observed between oxidative stress and muscle mass [107–110]. The disruption of signaling pathways involving skeletal muscle reactive oxygen species has received increasing attention [6]. Age-associated accumulation of nitrotyrosine in muscle proteins was reported [108]. The accumulation of mitochondrial and nuclear DNA damage leads to the loss of skeletal muscle fibers [111]. Mitochondria-mediated apoptosis represents a central process driving age-related muscle loss [112]. Mitochondrial dysfunction is related not only to the loss of its capacity to generate ATP but also to the activation of apoptotic pathways leading to the irreversible cell loss that is characteristic of sarcopenia [112].

Further studies have shown that ROS accumulation can increase proteolysis leading to loss of muscle mass; increased ROS production activates the ubiquitin-proteasome pathway. Aging is associated with greater proteasome content and activity [113], increased expression of the ubiquitin ligase MuRF1 (Muscle RING-finger protein-1) and atrogin-1 [114], and increased calpain activity [115]; however, further studies are required to explore the role of oxidative stress in these age-related alterations. The potential role of age-dependent mitochondrial dysfunction and cumulative oxidative stress as the underlying cause of age-associated fiber atrophy remains controversial; the pharmacological attenuation of age-related mitochondrial redox changes failed to rescue the age-associated muscle fiber atrophy, implying that the muscle mitochondrial redox environment is not a key regulator of fiber atrophy during sarcopenia [3].

Recently it was reported that sedentary humans display an age-related decline in the mitochondrial protein optic atrophy 1 (OPA1) that is associated with muscle loss [116]. FoxOs (Forkhead box proteins) are master regulators of autophagy and the ubiquitin-proteasome system [117] and are activated by oxidative stress and Akt inhibition. Importantly, the acute inhibition of OPA1 results in an increased oxidative stress, and *in vivo* inhibition of FoxOs was sufficient to reduce muscle atrophy in *Opa1*^−/−^ mice [117].

## 6. Antioxidant Therapies and Effects of Exercise on the Aged Muscle

The effects of exercise on aging in the skeletal muscle are very controversial. There is widespread agreement that oxidation could increase during exercise. Early studies have suggested that ROS play important roles in the inflammatory response to high-intensity or long-lasting exercise [118]. On the other hand, it has also long been known that moderate exercise increases the antioxidant capacity of the skeletal muscle by mitochondrial remodeling [119]. A more recent study suggested that endurance training stimulates mitochondrial remodeling which leads to an increase in mitochondrial content and function [120]. Unfortunately recent rodent models suggest that exercise-induced mitochondrial remodeling is defective in the aged muscle [121]. In contrast, as mentioned above, moderate exercise can improve the number of CRU-mitochondria pairs and thus provide more ATP and Ca^2+^ for contraction [68]. Furthermore, resistance-type exercise training represents an effective strategy to increase satellite cell content and reverse type II muscle fiber atrophy in humans [81].

Another target of exercise against oxidative stress is the increased activity of enzymatic antioxidants (i.e., glutathione peroxidase, catalase, and superoxide dismutase) accompanying the exercise-induced ROS generation. For example, skeletal muscle-specific manganese superoxide dismutase-deficient mice, which showed reduced exercise activity without atrophy, presented significantly improved exercise activity of the skeletal muscle after a single administration of an antioxidant [122].

Numerous investigations have aimed to explore the effects of antioxidant treatment on skeletal muscle performance. Some of them also studied old muscles and found positive effects of such a treatment. For example, hydroxytyrosol, which has high free-radical-scavenging capabilities, caused increased *in vivo* force in aged rats [123]. Recent studies showed positive effects of resveratrol [124], some plant extracts (*Rhus coriaria* [125] and *Rosmarinus officinalis* [126]), and vitamins (vitamin C [127]). The increasing number of similar studies nowadays shows the importance and topicality of finding good antioxidant treatment for the aged muscle.

As discussed above, several studies report an elevation in levels of oxidized protein and DNA in the older skeletal muscle. To date, RyR is the only key protein in EC coupling for which lifelong voluntary training was investigated and found to improve its expression level in aged mice (unpublished data of the authors). The data showed a beneficial antioxidant effect of selenium supplementation on skeletal muscle performance in old animals. However, as the authors could not prove the direct effects of antioxidant treatment on ROS production, there are several key proteins in EC coupling which could be positively altered and, thus, enhance force production.

The effect of antioxidant compounds on aged satellite cells has already been reported. Tocotrienol is a vitamin E analogue bearing high antioxidant activity. The tocotrienol-rich fraction (TRF) replenished the regenerative capacity of the human senescent satellite cells [128]; furthermore, TRF is able to ameliorate antioxidant defence mechanisms and improve replicative senescence-associated oxidative stress in human satellite cells [129]. The vitamin E analogue trolox treatment prevented the appearance of senescence markers, restored the expansion, and rescued the proliferative and regenerative defect of geriatric satellite cells [72]. The effect of resveratrol was studied in the mouse myoblast cell and showed protection against ROS by improving Sirt1 (Sirtuin1) levels, increasing antioxidant production, and reducing apoptotic signaling and cell death [130]. Interestingly, the effect of exercise on the oxidative stress of satellite cells has not yet been investigated in the literature. The protective effects of exercise, resveratrol, and their combination was shown to increase muscle mass in rats, probably associated with antiapoptotic signaling pathways through activation of AMPK (AMP-activated protein kinase)/Sirt1 [131]. In contrast, administration of the long-term mitochondria-targeted antioxidant, mitoquinone mesylate, failed to attenuate age-related oxidative damage or rescue the loss of muscle mass and function in the skeletal muscle of old mice [132].

## 7. Concluding Remarks

Lifelong maintenance of muscle mass and strength is a global health challenge. With an aging population, the problem of sarcopenia is becoming more and more important, and effective strategies are required to improve muscle performance. An average 30-year-old will lose about 25% of his or her muscle strength by age 70 and 50% of it by age 80. The improvement of mobility and independence is key for old people, and it relieves society from healthcare and social support costs. Our knowledge about the signaling pathways mediating age-related muscle loss is still limited. Oxidative stress and subsequent alterations in signaling pathways could lead to different pathophysiological events at different stages of life, especially in old age. As was shown in this review, a lot of targets in skeletal muscle could be altered by increased oxidative stress with aging. Some of them are targets of intrinsic factors, but there are some which depend mainly on extrinsic actions. The effects of oxidative stress in muscles are so diverse that improving only one step is usually not enough to get better muscle performance. This means that only combined therapy could be effective, and continuous training will also allow muscle fibers to incorporate higher levels of exogenous antioxidants from dietary supplements.

In conclusion, the risks of oxidative stress-induced damage can be minimized with regular exercise, which has beneficial effects on physical and mental health. We have to emphasize that while it is never too late to begin exercise, an early start and regular practice throughout life would greatly improve outcomes in later years and slow down a body's aging process. A lot of people try to start a training program late in life, when muscle performance is already diminished. It follows that muscle research has to promote the development of a new generation of physically active, healthy elderly citizens. To achieve this, and to minimize oxidative stress, the key could be a carefully developed exercise protocol combined with adequate antioxidant supplementation. However, exercise can be restricted due to orthopedic or cardiopulmonary limitations, which highlights the importance of the exploration of antioxidant therapy or nontraditional exercise. Regular exercise to maintain muscle function also has beneficial effects by reducing oxidative stress, not only in the muscle, but in all tissue, a fact that intrinsically would reduce/delay aging.
